# Antimicrobial Peptides Pom-1 and Pom-2 from *Pomacea poeyana* Are Active against *Candida* *auris*, *C. parapsilosis* and *C. albicans* Biofilms

**DOI:** 10.3390/pathogens10040496

**Published:** 2021-04-20

**Authors:** Heinz Fabian Raber, Jetmira Sejfijaj, Ann-Kathrin Kissmann, Andreas Wittgens, Melaine Gonzalez-Garcia, Annia Alba, Antonio A. Vázquez, Fidel E. Morales Vicente, Julio Pérez Erviti, Dennis Kubiczek, Anselmo Otero-González, Armando Rodríguez, Ludger Ständker, Frank Rosenau

**Affiliations:** 1 Institute of Pharmaceutical Biotechnology, Ulm University, 89081 Ulm, Germany; Heinz.Raber@uni-ulm.de (H.F.R.); jetmira.sejfijaj@uni-ulm.de (J.S.); ann-kathrin.kissmann@uni-ulm.de (A.-K.K.); a.wittgens@gmx.de (A.W.); dennis.kubiczek@gmx.com (D.K.); 2 Center for Protein Studies, Faculty of Biology, University of Havana, 25 Street, Havana 10400, Cuba; mgonzalez@fbio.uh.cu (M.G.-G.); julio.perez@fbio.uh.cu (J.P.E.); aotero@fbio.uh.cu (A.O.-G.); 3 Pedro Kourí Institute for Tropical Medicine, Havana 13600, Cuba; annia.alba@gmail.com (A.A.); antonivp@ipk.sld.cu (A.A.V.); 4 General Chemistry Department, Faculty of Chemistry, University of Havana, Zapata y G, Havana 10400, Cuba; femvicente@gmail.com; 5 Synthetic Peptides Group, Center for Genetic Engineering and Biotechnology, P.O. Box 6162, Havana 10600, Cuba; 6 Core Facility for Functional Peptidomics, Faculty of Medicine, Ulm University, 89081 Ulm, Germany; armando.rodriguez-alfonso@uni-ulm.de (A.R.); ludger.staendker@uni-ulm.de (L.S.); 7 Max Planck Institute for Polymer Research Mainz, Ackermannweg 10, 55128 Mainz, Germany

**Keywords:** antimicrobial peptide, *Candida* species, biofilm

## Abstract

Recently two peptides isolated from the Cuban freshwater snail *Pomacea poeyana* (Pilsbry, 1927) were described to have antimicrobial activity against bacterial pathogens. Here we show considerable activities of Pom-1 and Pom-2 to reduce the viability of *C. albicans*, *C. parapsilosis* and the less common species *C. auris* measured as the decrease of metabolic activity in the resazurin reduction assay for planktonic cells. Although these activities were low, Pom-1 and Pom-2 turned out to be highly potent inhibitors of biofilm formation for the three *Candida* species tested. Whereas Pom-1 was slightly more active against *C. albicans* and *C. parapsilosis* as representatives of the more common *Candida* species Pom-2 showed no preference and was fully active also against biofilms of the more uncommon species *C. auris*. Pom-1 and Pom-2 may represent promising lead structures for the development of a classical peptide optimization strategy with the realistic aim to further increase antibiofilm properties and other pharmacologic parameters and to generate finally the first antifungal drug with a pronounced dedication against *Candida* biofilms.

## 1. Introduction

Although generations of microbiologists regarded the traditional microbiological cultivation in Erlenmeyer-flasks as the most preferable condition for their research organisms, biofilms formed on different materials as substrates are by far the most relevant and the “normal” lifestyle for microorganisms in general [1]. This is in principle also the case for all relevant pathogenic bacteria and lower eukaryotes. Estimations exists that biofilms may be associated with 65% of hospital acquired infections and may account for more than 80% of all microbial infections [2,3,4]. Almost undisputed is a key feature of microbial biofilms which has been recognized as a significant increase of resistance to chemical and physical stresses, qualifying them as a major thread in clinical environments and their treatment as a socio-economical challenge for health systems [5,6]. Fungi of the genus *Candida* represent a class of highly important pathogens with the commensal yeast *C. albicans* probably being the most abundant and common species in clinical fungal infections [7]. In their life a majority of women of 75% will suffer from Candidiasis and 85–95% of these infections in this group of patients are caused by *C. albicans* [8]. However the leading role of *C. albicans* in invasive infections is decreasing in the last decades and other *Candida* species increase in accounting for infections [9,10,11]. Among them *Candida parapsilosis* is relevant, as it is able to form persistent biofilms on catheters, medically implanted devices and all abiotic or biotic surfaces in medical care units, thus posing severe threads to the patients especially after invasive surgery. In addition, *C. parapsilosis* is perfectly nourished by total parenteral nutrition of patients, thereby representing a more then considerable risk for undernourished children and low-birth-weight neonates [9,11,12,13,14]. Furthermore, several clinical isolates of this species have been reported to lose susceptibility to important classes of antifungal drugs, which poses severe limitations to efficient treatment regimes [15,16,17,18].

**Figure 1:**
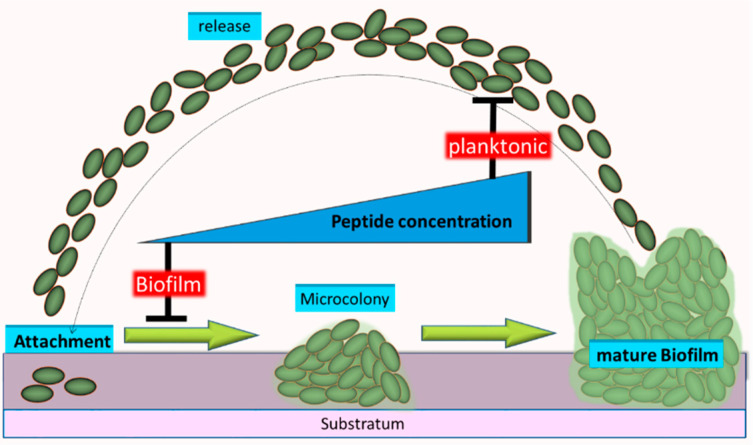
Schematic overview of a typical Candida biofilm and stages of action for Pom-1 and Pom-2 on biofilm development. The simplified biofilm formation process can be divided into four phases, (i) the attachment of planktonic cells to the future biofilm substratum, (ii) the aggregation of cells and their subsequent growth into microcolony like structures and (iii) the development of species-specific mature biofilm architectures. (iv) Release of cells into the planktonic phase delivers Candida seeds for the next generation of biofilms. Already low concentrations of Pom-1 and Pom-2 can inhibit the development of biofilms, whereas only unreasonably high concentration of the peptide is required to affect the viability of planktonic cells.

*Candida auris*, however, is a more recently discovered pathogenic yeast and thus probably one of the most uncommon *Candida* which was first isolated in 2009 from a Japanese patient [19]. *C. auris* can cause severe bloodstream infections in hospitalized patients and can lead to remarkably high mortalities between 35% and 60% [20,21]. Special threat arises from the fact that strains of *C. auris* with multiple drug-resistances (probably by upregulation of ABC-type efflux pumps [22]) against prominent antifungal drugs have occurred independently in different countries/continents worldwide [23]. Thus, both, the U.S. center for disease control (CDC) and the European center for disease control (ECDC) have released clinical alerts, initiating also a broad public discourse, identifying *C. auris* as an emerging “superbug” [24]. Multi-drug resistance of *C. auris* against classical antifungal drugs like fluconazole or amphotericin B has been discussed to be caused by the activity of ABC-type MFS (major facilitator superfamily) efflux pumps [22], which are even overexpressed in *C. auris* when living in biofilms [25]. Based on these reports, biofilm formation and the resulting increase of physiological robustness can be regarded as a key feature of virulence by common, as well as by uncommon *Candida* species to successfully establish their full pathogenic potential in combination with resistance against classical fungicides. Currently, no biofilm-specific drugs exist today for *Candida* species or any other microbe, making treatment of biofilm-based infections particularly problematic [26]. In our opinion, systematic studies to isolate specific anti-biofilm drug molecules against *Candida* species are currently far from being common in the scientific community but they are urgently needed. They could represent the scientific foundation for the development of a completely new piece of munition in the fight against pathogenic microbes in general based on the novel activities against their biofilms. Simplified to the extreme the formation of elaborate microbial biofilms can be divided into only four phases, the attachment of planktonic cells to the future biofilm substratum, the assembly of cells and the subsequent growth into microcolony like structures, the development of species-specific mature architectures of the biofilm and finally the release of the previous biofilm cells into the planktonic phase (Figure 1).

One promising class of new therapeutic molecules not only in the fight against planktonic *Candida* cells but also against biofilms of these pathogenic yeasts are antimicrobial peptides (AMPs). Most AMPs have a simple physical mode of action reducing the functional integrity of microbial cell membranes. They act probably by forming “barrel stave” like [27,28,29], “toroidal” like [29,30,31] or “carpet” like [29] pores or related activities [32,33] and can be considered to provide excellent treatment options even against organisms resistant to conventional antifungal drugs. The AMP Cm-p5 derived from a natural peptide originally isolated from the coastal mollusk *Cenchritis muricatus* exhibited efficient antifungal activity against different fungal pathogens [34] including *C. auris* [35,36], with only neglectable cytotoxic effects towards mammalian cells [34]. Interestingly, synthetic dimeric derivatives of Cmp-5 exhibited a considerable activity towards *C. auris* biofilms on an abiotic substratum inhibiting the growth of biofilms but had only low activities against the respective planktonic cells compared to the original Cm-p5 [37]. Recently we have described another set of AMPs isolated from the Cuban freshwater snail *Pomacea poeyana* (Pilsbry, 1927) [38]. These two peptides named Pom-1 and Pom-2 demonstrated high antimicrobial activity against the Gram-negative bacteria such as *Pseudomonas aeruginosa* while it demonstrates only a moderate activity against *Klebsiella pneumoniae* and *Listeria monocytogenes* in combination with low toxicity towards primary human macrophages [38].

Here we show considerable activities of Pom-1 and Pom-2 to reduce the viability of *C. albicans*, *C. parapsilosis* and the less common species *C. auris* measured as the decrease of metabolic activity in the resazurin reduction assay for planktonic cells [39]. Although these activities can only be considered to be at least limited Pom-1 and Pom-2 turned out to be highly potent inhibitors of biofilm formation for the three *Candida* species tested. Whereas Pom-1 was slightly more active as compared to Pom-2 against *C. albicans* and *C. parapsilosis* as representatives of the more common *Candida* species Pom-2 showed no preference and was fully active also against the uncommon species *C. auris*. Pom-1 and Pom-2 may represent promising lead structures for the development of a classical peptide optimization strategy with a realistic aim to further increase antibiofilm properties and other pharmacologic parameters and to isolate finally the first antifungal drug with a pronounced dedication against *Candida* biofilms.

## 2. Results and Discussion

**Figure 2:**
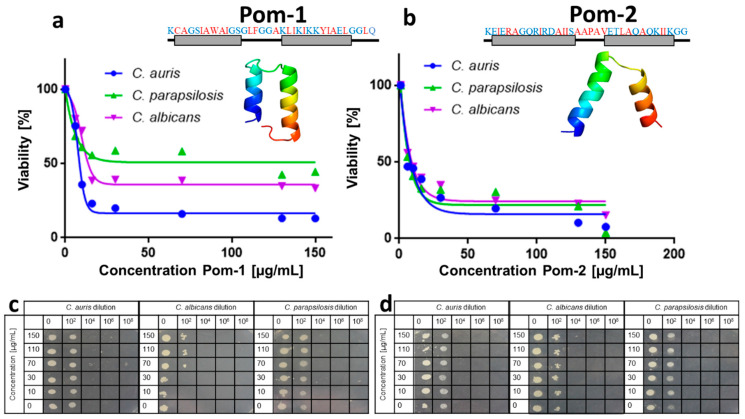
Action of Pom-1 and Pom-2 on the *Candida* species. (**a**,**b**) Pom-1 and Pom-2 dose-dependent activity of *Candida* species viability determined by the resazurin reduction test. All experiments were performed in triplicates. Standard deviations have been calculated and found to be too low to be visible in the graphs. Modelled 3D-structure of Pom-1 and Pom-2 using the QUARK and SwissModel server and the corresponding amino acid sequence [38]. The red letters represent hydrophobic amino acids and the blue hydrophilic. Grey boxes represent alpha-helix structures. (**c**,**d**) plate-spot assay for Pom-1 and Pom-2 measuring antifungal activities against dilutions of *C. auris*, *C. albicans* and *C. parapsilosis*. Colony formation was monitored after 24 h of growth in the presence of increasing concentrations in 10^2^, 10^4^, 10^6^ and 10^8^ dilutions of the original cultures, which were adjusted to identical optical densities prior peptide addition. Dilutions were then spotted on agar plates and further incubated for another 24 h at 37 °C.

Pom-1 is a 34 amino acid peptide comprising a predicted structure of two alpha helices connected by a 6 aa loop (Figure 2a), whereas Pom-2 has a sequence length of 33 aa also delivering a predicted secondary structure of two alpha helices joint in this case by only three amino acids (Figure 2b). 

### 2.1. Activity against Planktonic Cells

Antifungal testing was done in this study by an assay combining the classical susceptibility test according to the Clinical and Laboratory Standards Institute (NCCLS) M27-A2 approved standard protocol as a reference method for broth dilution antifungal susceptibility testing of yeast [40] with rapid resazurin based measurements of cell viability for assessing the toxicity of fungicides [39] thereby replacing the simple optical inspection and ranking of turbidity by the more sensitive fluorescence measurement of resorufin in the latter assay. In this assay performed with planktonic cells of the respective yeasts the peptide Pom-1 delivered semi-inhibitory concentrations for planktonic cell viability (_IC50P_) values of 8.5 µg/mL for *C. auris* 13.8 µg/mL for *C. albicans* and 36.9 µg/mL for *C. parapsilosis* with a generally only moderate activity but with a slight preference for *C. auris* (Figure 2a,c). With IC_50P_ values of 8.4 µg/mL for *C. auris* 9.3 µg/mL for *C. albicans* and 7.5 µg/mL for *C. parapsilosis* the overall activity of Pom-2 was comparable but with almost identical courses of the curves (Figure 2b,d). Although the IC_50P_ values appeared to suggest promising activities for both peptides, the inhibition was incomplete with viabilities approximating 13% for Pom-1 and 8% for Pom-2 for *C. auris* as the most susceptible species in our set of pathogenic yeasts even at very high drug concentrations of 150 µg/mL. 

### 2.2. Anti-Biofilm Activity

Only moderate activities against planktonic cells has also been described for a set of derivatives a snail derived natural antimicrobial peptide, which turned out to possess remarkable capabilities to inhibit the biofilm formation of *C. auris* probably based on a yet so far uncharacterized additional activity at very low concentrations of the peptides [37].

The inhibiting activity of classical antimicrobial peptides is directed (and usually characterized) against free-floating planktonic cells. However, probably the more important microbial lifestyle in fact can be considered to be the biofilm, especially in the case of pathogenic microbes during their interaction with host tissues deciding their success to establish their full pathogenic potential in the onset of an infection [4,6,26,41,42]. Although the importance of anti-biofilm drugs has already been recognized in the early times of biofilm research dedicated compounds against *Candida* species or any other microbe have been and are still awaiting their discovery keeping treatment of biofilm-based infections particularly problematic [26]. 

**Figure 3:**
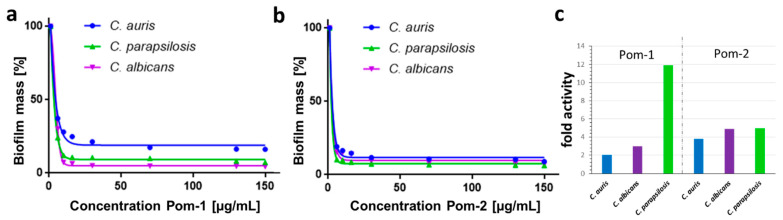
Biofilm inhibition by the peptides Pom-1 and Pom-2 (**a**,**b**) dose-dependent inhibition of *C. auris*, *C. parapsilosis* and *C. albicans* de novo biofilm formation by Pom-1 and Pom-2 quantified with crystal violet after 24 h in triplicates. All experiments were performed in triplicates. Standard deviations have been calculated and found to be too low to be visible in the graphs. Inhibitors were present throughout the growth. (**c**) Demonstration of the higher activity in biofilm inhibition of Pom-1 and Pom-2 by comparing the ratio of the IC_50b_ and IC_50p_ values on the Candida species.

The activity against planktonic cells was at most moderate, however, when Pom-1 and Pom-2 were tested in an experimental setup in which cells were allowed to form biofilms on the polystyrene surface of microtiter plates in the presence of increasing concentrations of the peptides both showed the desired antibiofilm activity. The development of biofilms was drastically reduced for all *Candida* species. The peptide Pom-1 showed a slight preference towards *C. albicans* and *C. parapsilosis* leading to a reduction of biofilm mass to 12% and 7% already at concentrations of 10 µg/mL whereas the susceptibility of *C. auris* diminished resulting in a biofilm reduction to only 28% for this concentration. Interestingly, in contrary, in the viability assay Pom-1 performed best against *C. auris* planktonic cells. This finding is a remarkable parallel to derivatives of a peptide from the coastal mollusk *Cenchritis muricatus* which also failed as antimicrobial peptides against *C. auris* planktonic cells but efficiently could inhibit biofilm formation [37]. Nevertheless, the respective semi-inhibitory concentration for biofilm inhibition (IC_50 Biofilm_) of Pom-1 were 4.2 µg/mL for *C. auris*, 4.6 µg/mL for *C. albicans* and 3.1 µg/mL for *C. parapsilosis* (Figure 3a). The second peptide Pom-2 also inhibited biofilm formation of all *Candida* sp. with IC_50B_ of 2.2, 1.9, 1.5 µg/mL for *C. auris*, *C. albicans* and *C. parapsilosis,* but unlike Pom-1 it lacked the difference between the efficiencies against individual species and was fully active also against *C auris* with reductions of the final biofilm masses down to 16% for *C. auris*, 15% for *C. albicans* and 10% for *C. parapsilosis* at peptide concentrations of 10 µg/mL (Figure 3b). Both, Pom-1 and Pom-2 appear to be more active in inhibiting biofilm formation than in reducing the viability of planktonic cells which became manifest in the comparison of the respective IC_50_ values for both processes. Consistently the activities against biofilms were elevated for all *Candida* species with a minimum of a 2-fold higher Pom-1 activity against *C. auris* biofilms up to a 12-fold increase for Pom-2 against *C. parapsilosis* (Figure 3c). 

The inhibitory effects of both peptides, Pom-1 and Pom-2, on early-stage biofilms were unexpectedly pronounced and positive especially with respect to their extremely limited activity towards planktonic cells. The molecular mode of action needs to be further elucidated for Pom-1 and Pom-2 to define to which already known models of pore formation [31] they belong. Nevertheless, one possible explanation for the observed biofilm preference or potentially existing specialized activity on biofilm cells may arise from the so-called charged lipid clustering. In this model of helical peptides accumulate at the surface of membranes and induce clustering of anionic lipids resulting in membrane depolarization and the slow leakage of intracellular content [31,43]. As a side effect of this peptide accumulation on cell surfaces and the alterations in charge distribution drastic effects on the physico-chemical properties of the cell surface with consequences for early events in biofilm formation like cell aggregation, their attachment to the substratum and their growth into microcolonies must be expected. This would probably allow developing optimized peptides with pronounced anti-biofilm properties as lead structures for a potentially new class of specific anti-biofilm drugs. An interesting study in this context identified a peptide from the South American rattlesnake *Crotalus durissus terrificus* which combined activities against planktonic cells of *Candida albicans* and the respective biofilms [44]. The findings presented here and their possible explanations may in fact qualify Pom-1 and Pom-2 as interesting molecules with a potential novel mode of action and specificity towards biofilms and thus as valuable targets for future in-depth studies to elucidate their properties and working mechanism. Interesting key questions are, then, how the sequence and the resulting structure of the peptides influence their distinct different activities towards planktonic cells and biofilms which can be addressed by repetitive synthesis of sequence variants, subsequent activity testing in combination with biophysical measurements of their interactions with bio-membranes and the consequences of these interactions for membrane (and thus cell) integrity.

## 3. Conclusions

The antimicrobial peptides Pom-1 and Pom-2 from the Cuban freshwater snail *Pomacea poeyana* (Pilsbry, 1927) have been shown to moderately reduce viability of *C. auris*, *C. albicans* and *C. parapsilosis* cells. Despite these limited activities against planktonic cells both peptides were potent inhibitors of biofilm formation with IC_50_ values between 1.5–4.6 µg/mL. This may qualify the peptides as lead structures for the first biofilm specific fungicide in the future.

## 4. Materials and Methods

### 4.1. Cultivation of Candida Species

*Candida auris* was purchased from DSMZ (DSMZ-No. 21092), *C. albicans* (ATCC 90028) and *C. parapsilosis* (ATCC 22019) were obtained from the Laboratory of Medical Mycology, IPK. The *Candida* species grown on YPD Agar (1% *w*/*v* yeast extract, 2% *w*/*v* peptone, 2% *w*/*v* glucose, 1.5% Agar). For suspension cultures 10 mL YPD medium in a 100 mL Erlenmayer flask was inoculated with a single colony and grown at 37 °C and orbital shaking at 150 rpm. Agar plates containing glucose (40 g/L), peptone (10 g/L) and agar (15 g/L) and were adjusted to a pH of 5.6.

### 4.2. Peptide Synthesis

Peptides were Produced by Solid Phase Synthesis as described in Gonzalez Garcia et al., 2020 [38].

### 4.3. Viability Tests and Quantification

For *Candida* species, the minimal inhibitory concentration (MIC) of Pomacea 1 and 2 and the viability of the yeasts was determined according to the “Clinical and Laboratory Standards Institute” guidelines M27-A3 broth microdilution assay. In brief, 2.5 × 10^3^ yeast cells were seeded in 200 µL RPMI-1640 medium supplemented with L-glutamine in a flat bottomed, 96-well polystyrene microtiter plates (Sarstedt AG & Co. KG, Nümbrecht, Germany) and incubated at 37 °C with agitation at 900 rpm on an Eppendorf shaker. The effect of the different Pomacea derivatives Pom-1 and Pom-2 on the cell viability was tested in the presence of the peptides at different concentrations. The cell viability was quantified by a resazurin assay according to Patricia Bi Fai et al. [39]. The cells were incubated with 100 µL resazurin with a concentration of 12 µg/mL for 40 min. Viable cells reduces resazurin to the fluorescent resorufin by the production of NADPH. The amount of produced resorufrin was analyzed by fluorescence measurements at excitation wavelength of 535 and an emission of 595 nm with a Tecan infinite M200 microplate reader to quantify the viability. The curve was fitted by a non-linear regression with dose-response nonlinear Hill equation. The semi-inhibitory concentration of the planktonic cell viability (IC_50p_) represents the point at which the viability of the cells is reduced to 50% compared to the untreated control. After the incubation 10 µL samples were collected for a plate spot assay. Therefore, a dilution row of each sample was performed in RPMI 1640 medium and 3 µL microliters of the dilutions were spotted onto Sabouraud agar plates. The agar plates were incubated for 24 h at 37 °C. The assay was performed according to the method described in Hilgers et al. [45].

### 4.4. Biofilm Formation and Quantification/Anti-Biofilm Treatment

Biofilms were basically formed and analyzed as described previously [46,47,48]. In brief, 2.5 × 10^3^ yeast cells were seeded in 200 µL RPMI-1640 medium supplemented with L-glutamine in a flat bottomed, 96-well polystyrene microtiter plates (Sarstedt AG & Co. KG, Nümbrecht, Germany) and incubated at 37 °C without agitation for 24 h. The effect of the different *Pomacea* on the biofilm formation was tested in the presence of Pom-1 and Pom-2 at different concentrations. The biofilm was quantified by a crystal violet assay, which was originally developed for bacteria by George O’ Toole [47,49] and is also widely used for *Candida* biofilms [46,48,50,51]. Planktonic cells were removed with the supernatant and the mature biofilms were washed twice with 200 µL water. Subsequently biofilms were stained with 200 µL of a 0.1% (*w*/*v*) crystal violet solution for 15 min. The supernatant was removed, and the biofilms were washed twice with 200 µL water to get rid of excess crystal violet. The stained biofilms were air dried for 24 h at 25 °C and finally destained using 200 µL of 30% acetic acid (15 min, 25 °C). The supernatant was transferred to a fresh 96 well plate and the absorbance at 560 nm was measured using a Tecan infinite M200 microplate reader to quantify the biofilm biomass. The curve was fitted by a non-linear regression with dose-response nonlinear Hill equation. The semi-inhibitory concentration of biofilm formation (IC_50b_) represents the point at which the biofilm mass is reduced to 50% compared to the biofilm mass of untreated control.

## Data Availability

Not applicable.
